# A data-driven approach to define the stages and patterns of neurological recovery after traumatic spinal cord injury

**DOI:** 10.1007/s00415-026-14065-9

**Published:** 2026-08-19

**Authors:** Wanda Lauth, Abel Torres-Espin, Angel Arevalo-Martin, Daniel Garcia-Ovejero, Orpheus Mach, Doris Maier, Rainer Abel, Norbert Weidner, Yorck-Bernhard Kalke, Jiri Kriz, Armin Curt, EMSCI Study Group, Catherine Jutzeler, Iris Leister

**Affiliations:** 1 Spinal Cord Injury Center, BG Trauma Center Murnau, Murnau, Germany; 2 Team Biostatistics and Big Medical Data, IDA Lab Salzburg, Paracelsus Medical University, Salzburg, Austria; 3 Research Programme Biomedical Data Science, Paracelsus Medical University, Salzburg, Austria; 4 School of Public Health Sciences, Faculty of Health, University of Waterloo, Waterloo, Canada; 5 Laboratory of Neuroinflammation, Hospital Nacional de Parapl´ejicos, SESCAM, Toledo, Spain; 6 Neuroinflammation Group, Instituto de Investigacion Sanitaria de Castilla-La Mancha (IDISCAM), Toledo, Spain; 7 Spinal Cord Injury Center, Klinikum Bayreuth, Bayreuth, Germany; 8 Medical Faculty Heidelberg, Heidelberg University, Heidelberg, Germany; 9 Spinal Cord Injury, Heidelberg University Hospital, Heidelberg, Germany; 10 Spinal Cord Injury Center, University Hospital Ulm, Ulm, Germany; 11 Spinal Cord Unit, University Hospital Motol and Homolka, Prague, Czech Republic; 12 Spinal Cord Injury Center, Balgrist University Hospital, Zurich, Switzerland; 13 Department of Health Sciences and Technology (D-HEST), ETH Zurich, Zurich, Switzerland; 14 University Clinic for Anesthesiology and Critical Care Medicine, Kepler University Hospital, Johannes Kepler University Linz, Linz, Austria

**Keywords:** Spinal cord injuries, Neurological recovery, Data-driven analysis, EMSCI

## Abstract

**Background:**

Recovery after spinal cord injury (SCI) follows a complex and variable trajectory, yet the field lacks clear, data-driven definitions of the temporal stages of recovery. This study aimed to model the trajectory of recovery after SCI and define distinct post-injury phases based on real-world clinical data.

**Methods:**

We analyzed longitudinal data from 4407 individuals with traumatic SCI enrolled in the European Multicenter Study about Spinal Cord Injury (EMSCI). Neurological improvement was quantified using the Integrated Neurological Change Score (INCS). Functional recovery was assessed using the Spinal Cord Independence Measure (SCIM) and a derived measure of hand motor function based on motor scores of the C6, C8, and T1 myotomes. Longitudinal recovery trajectories were modeled using random forest regression, locally estimated scatterplot smoothing (LOESS), and neural networks. Lastly, latent class mixed models (LCMM) were employed to identify subgroups with distinct recovery profiles.

**Results:**

Across all models, neurological recovery followed a four-stage sigmoidal pattern: an acute phase (up to ∼2 weeks post-injury) with minimal change; a transition phase (∼2 to 9–11 weeks) marked by substantial neurological change; a pre-chronic phase (∼10 weeks to 7–8 months) characterized by ongoing but non-linear recovery; and a chronic phase (beyond ∼7–8 months) in which recovery plateaued. Neurological improvement consistently preceded functional gains captured by SCIM and hand motor strength. Latent class analysis identified four distinct recovery profiles: class 1 (marked recovery), classes 2 and 3 (moderate recovery), and class 4 (minimal to no recovery). Notably, despite significant variability in the magnitude of lower extremity recovery across classes, the timing of the transition phase was remarkably consistent, aligning with the population-level window of approximately 10–11 weeks post-injury.

**Conclusions:**

The majority of neurological and functional improvement occurs within the first ∼10 weeks post-injury. These findings align with the concept of a period of heightened neuroplasticity and provide a data-driven framework for timing assessments and interventions in SCI recovery.

**Supplementary Information:**

The online version contains supplementary material available at https://doi.org/10.1007/s00415-026-14065-9.

## Background and rationale

Neurological recovery, defined by changes in neurological function across the various stages of injury, represents a critical endpoint in spinal cord injury (SCI) research. Given that injury severity is a key determinant of prognosis, the assessment and quantification of neurological function have been extensively studied.[1–4]. The International Standards for Neurological Classification of SCI developed by the American Spinal Injury Association (ISNCSCI) [5] are widely accepted for evaluating neurological function in SCI. However, their application in research settings presents limitations. In particular, the ASIA Impairment Scale (AIS) distinguishes complete (AIS A) from incomplete (AIS B–D) injuries solely based on sacral sparing, without accounting for residual sensory or motor function. As a result, critical information, such as potentially preserved sensory and motor function, is not considered, and the outcome primarily reflects the function of S4/S5. [6, 7]. Furthermore, using AIS grade conversion (defined as an improvement of one or more AIS grades between two time points) to quantify neurological improvement over time presents challenges due to ceiling and flooring effects making AIS conversion an insensitive measure of neurological change at both ends of the spectrum [8]. Also, in studies encompassing all AIS grades, the distribution is typically skewed, with AIS A representing the largest subgroup [9, 10]. AIS conversion also correlates poorly with functional outcomes such as walking ability and fails to capture clinically meaningful functional gains [11, 12]. Given these limitations, we therefore chose not to use AIS grade conversion as a primary endpoint in this study, in favor of more nuanced measures that better capture functional recovery.

To reliably measure changes in neurological function, two key components are needed: (1) an appropriate measure for quantifying neurological impairment, and (2) an understanding of the correct post-injury time frame for assessment. The first component has been extensively studied, with prior research examining changes in ISNCSCI sub-scores (motor and sensory) [4, 13–15], or functional outcomes such as walking ability [16–19]. Given the heterogeneity of SCI presentation, precise quantification of neurological change may benefit from composite endpoints derived from ISNCSCI sub-scores, supplemented by measures such as the Spinal Cord Independence Measure (SCIM) and other relevant clinical assessments, including segmental motor function.[8, 20]. The Integrated Neurological Change Score (INCS) is one such composite measure, ranging from –1 to +1, that provides a continuous metric capturing changes across sensory and motor domains based on ISNCSCI sub-scores [8].

The optimal timing for assessing neurological function to accurately evaluate recovery remains uncertain. Clinical studies require early and precise assessments, yet in routine practice ISNCSCI evaluations are often incomplete in the acute phase due to factors such as concomitant injuries, or limited patient compliance [21, 22]. As a result, our analyses rely on later measurements. It is unclear whether assessments conducted days or weeks after SCI onset still represent the acute neurological state without missing changes that may have occurred earlier [23]. Furthermore, there is no consensus on when SCI enters the chronic stage, where further significant neurological changes are unlikely [4, 9, 15, 24, 25].

The aim of this study was to evaluate time frames reflecting different stages of SCI (acute–transition–chronic) based on data from the European Multicenter Study on Human Spinal Cord Injury (EMSCI). By analyzing the change in neurological impairment over time, we aimed to assess 1) to what extent subsequent measurements (days or even weeks after injury) still reflect acute neurological impairment without missing changes that might have happened in the acute stages already and 2) when neurological recovery reaches a plateau (i.e., when the chronic stage of SCI is reached beyond which no further changes in neurological impairment are anticipated). Additionally, we aimed to 3) investigate whether distinct groups based on their recovery pattern, independent of the severity of the injury and the neurological level of the injury (NLI), could be derived from the data.

## Methods

We retrospectively analyzed neurological and functional outcome data from EMSCI to define post-injury time frames that distinguish the acute, subacute, transition, and chronic stages of SCI. To achieve this, we examined recovery trajectories using established clinical outcome measures for neurological and functional status. All analyses used days after injury ($t=\mathrm{D}\mathrm{A}\mathrm{I}$) as the continuous timing variable, independent of EMSCI-defined assessment windows, which were displayed only for context and not used for binning, staging, or modeling.

**Fig. 1:**
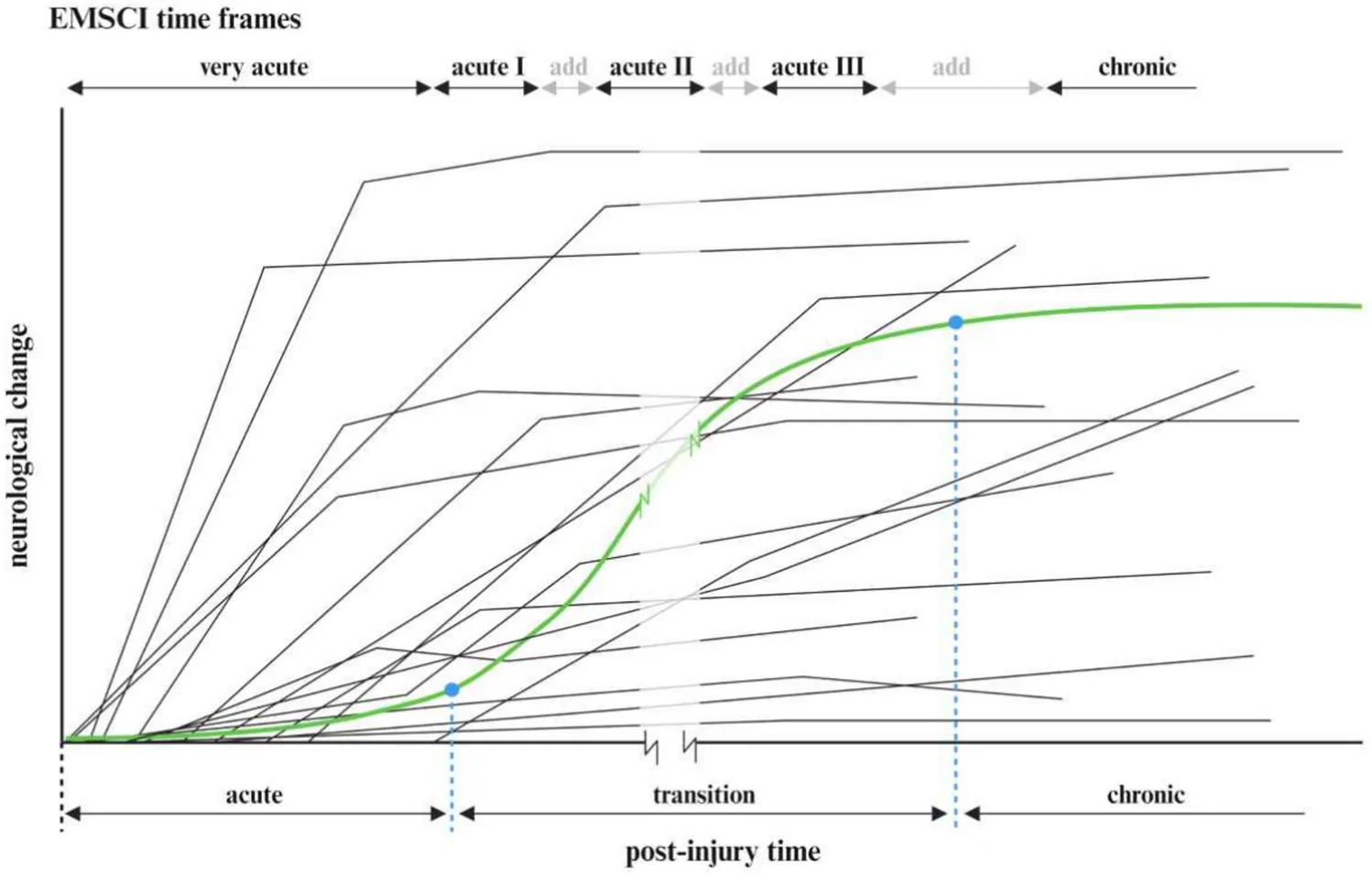
Stages of spinal cord injury based on the trajectory of neurological recovery: hypothetical individual recovery trajectories (black lines) and their expected trend (green curve) on a population level suggest a sigmoid pattern of neurological change over time. We hypothesized that the post-injury stages can be classified into acute, transition, and chronic phases. Vertical dashed lines indicate inflection points used to delineate these phases. The predefined EMSCI assessment time frames are depicted at the top of the figure: very acute = days 1–15; acute I = days 16–40; acute II = days 70–98; acute III = days 150–186; chronic = days 300–546; add = additional ISNCSCI assessments which do not fall into the planned EMSCI time frames. *EMSCI* European multicenter study on human spinal cord injury, *ISNCSCI* International standards for neurological classification of spinal cord injury

Based on prior evidence and clinical observations, we hypothesized that neurological recovery after SCI follows a sigmoid (’S-shaped’) trajectory (Fig. 1) at the population level, with the understanding that the timing and steepness of this trajectory differ across AIS grades and NLI: the first stage extends from the day of the injury until the neurological change curve begins to increase, indicating the acute injury stage when no significant functional changes are expected due to spinal shock and neuroinflammation among other mechanisms of secondary injury. The transition stage presents the period where most neurological change occurs, and, as such, it corresponds to the interval of the steepest slope within the curve. Lastly, the chronic stage begins as the neurological change curve begins to plateau. In this study, we first model the pooled trajectory as a population-level reference to derive data-driven global milestones of recovery, and then report stratified trajectories and parameters for AIS grades and NLIs to quantify grade-/level-specific timing and slopes.

### Data extraction and cleaning

**Fig. 2:**
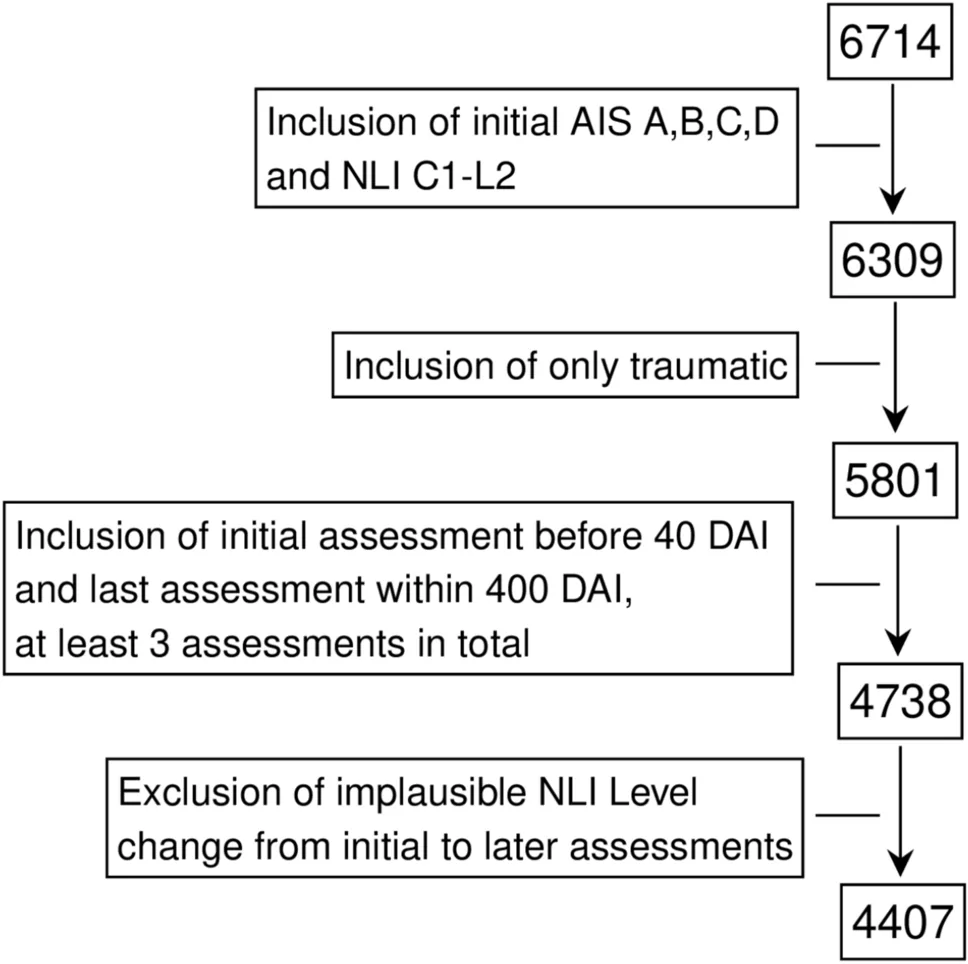
Inclusion and exclusion criteria with the respective change in sample size. *AIS* American Spinal Injury Association Impairment Scale, *NLI* neurological level of injury, *DAI* days after injury

For this study, we obtained access to the EMSCI dataset from the EMSCI study group, comprising ISNCSCI and SCIM scores collected between July 2001 and January 2023. To quantify neurological change, we calculated the INCSs between the first and all subsequent neurological assessments for each individual. The INCS was utilized to capture nuanced motor and sensory improvements while mitigating the ceiling and floor effects often associated with categorical measures such as the AIS [8]. To examine the trajectory of neurological change, INCS values were plotted against time in days (DAI), generating a scatterplot of all assessments and individual patient trajectories [9]. Assessment density was unevenly distributed across the post-injury timeline, with high-density regions corresponding to EMSCI examination periods and sparse intervals in the “additional” time frames (Fig. 1 and supplementary Fig. 9). A marked decline in assessment density was observed in the chronic stage. To reduce bias in trend estimation, we analyzed the temporal distribution of assessments and identified the DAI threshold at which density dropped below 5% of the peak amplitude within a 1-week observation window (Supplementary Fig. 10a). Assessments beyond 400 days post-injury were excluded. Evaluation of neurological function over time requires an initial reference measurement, ideally obtained at hospital admission. However, many patients had their first ISNCSCI assessment during or after the EMSCI “acute I” phase (days 16–40), as individuals are often first treated in a trauma hospital and only later transferred to an EMSCI-affiliated rehabilitation center, where ISNCSCI assessment data then become available for analysis. Beyond day 40, the density of first assessments fell below 5% of the peak amplitude within a 1-week window (Supplementary Fig. 10b). Therefore, one inclusion criterion, the initial ISNCSCI assessment within 40 days post-injury, was excluded (Fig. 11). Additionally, all observed patients have to had at least 3 assessments in total until 400 days after incident. It did not matter when the second assessment was conducted. While strict inclusion criteria were applied regarding timing of the initial assessment to ensure methodological consistency, we did not exclude outliers, as these data reflect real-world clinical variability. Removing patients with exceptionally good or poor outcomes would have limited representation of the full spectrum of recovery patterns. However, more than 10% of the remaining sample exhibited changes in NLI exceeding three segments over the observation period—magnitudes that are unlikely given typical recovery patterns and more consistent with measurement error or transient confounders at the initial exam (e.g., concomitant limb injuries, pain, or immobilization). To mitigate the impact of such likely misclassification, we applied a within-baseline-level outlier filter: for each initial NLI stratum (C1–C8, T1–T12, L1–L2), we calculated each patient’s maximum NLI shift over time and excluded the extreme 10%—specifically, the top 5% with the largest apparent improvements (caudal shifts) and the bottom 5% with the largest apparent deteriorations (rostral shifts)—relative to others with the same initial NLI. This leaves a total of 4407 patients and 15,191 data points (i.e., ISNCSCI assessments). The exact inclusion and exclusion criteria with the respective reduction in sample size are shown in Fig. 2.

### Modeling

We used three different machine learning-based methods for regression analysis, including estimated scatterplot smoothing (LOESS) [26–29], random forests [30], and neural networks [31]. Each algorithm and its associated regression curves were tuned through a hypergrid of selected hyperparameters [32]. A detailed description of hyperparameter tuning can be found in the supplementary material. To reduce fluctuations in the random forest models, a one-pass loess smoothing was applied upon their predictions, optimizing its span based on RMSE. For each regression method, 95% confidence bands were computed. For the random forest, bootstrap percentiles were used. For LOESS and the neural network, analytical confidence bands were applied; however, for the neural network, bootstrapping was employed to estimate the standard error. The bootstrapping procedures were based on 1000 resamples. All statistical analyzes were performed in R (version 4.3.2) [29].

#### Identifying stages

**Fig. 3:**
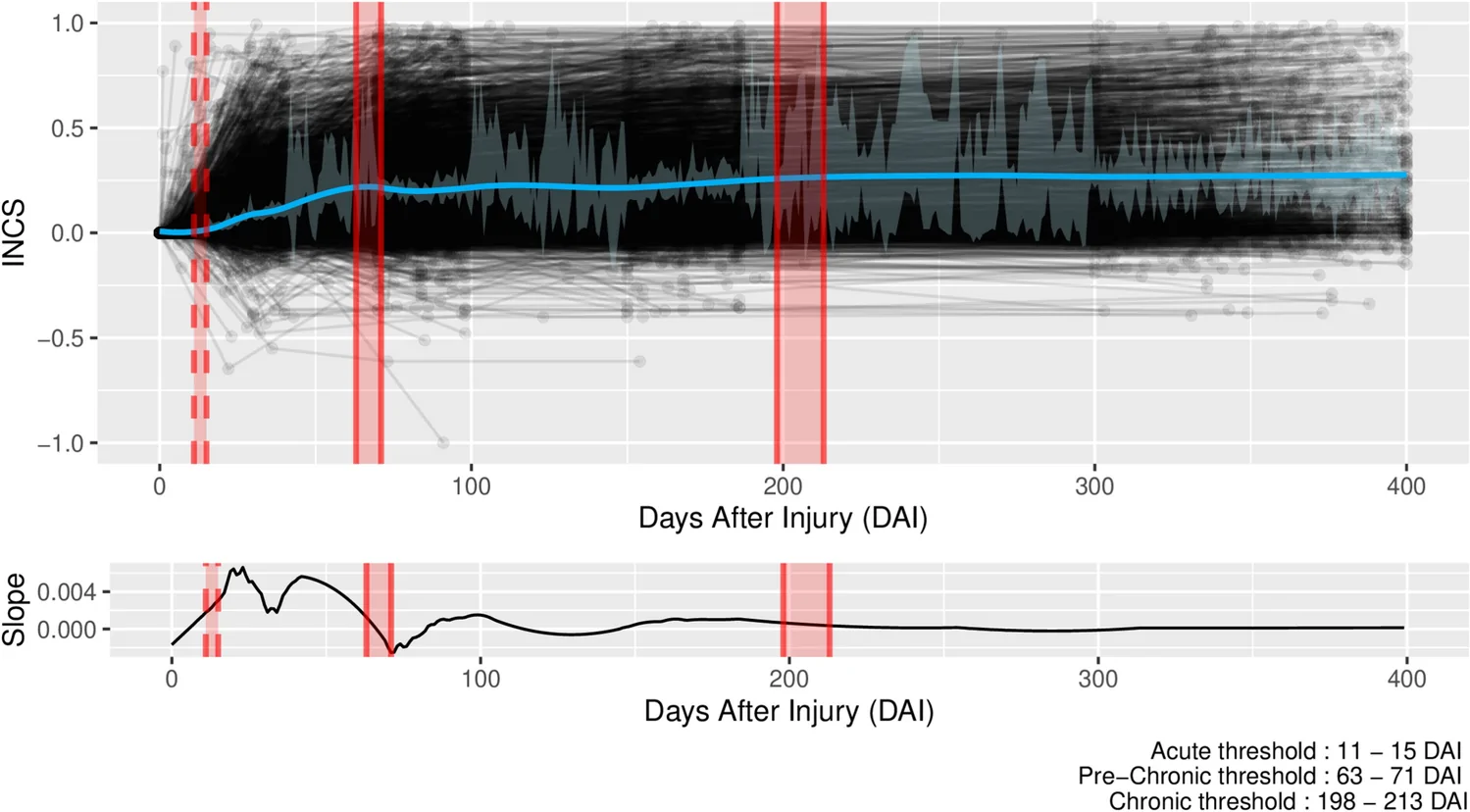
Neurological recovery trajectory based on INCS using random forest regression: scatterplot of all available INCS values (i.e., the change in neurological function from each patient’s first assessment to all subsequent assessments) plotted against days after injury, overlaid with the random forest regression curve (blue line). Dots represent individual neurological assessments, with lines connecting them to illustrate individual patient trajectories. The upper panel shows the estimated recovery trajectory, while the lower panel displays the first derivative (slope) of the smoothed curve, reflecting the rate of neurological change over time. Vertical lines (red) indicate the data-driven thresholds between recovery stages: acute-to-transition (DAI 11–15), transition-to-pre-chronic (DAI 63–71), and pre-chronic-to-chronic (DAI 196–208). *INCS* Integrated Neurological Change Score, *DAI* days after injury

As hypothesized, the regression analyzes yielded sigmoidal-shaped curves. However, instead of transitioning directly into a plateau after the steepest increase, a phase of slight fluctuating variations persisted for weeks before stabilizing in the chronic stage (see Fig. 3). This observation led us to revise the expected three-phase model and instead propose a four-phase trajectory: the acute phase, a transition phase with significant neurological change, a pre-chronic phase with non-linear variations, and the chronic phase where it plateaus.

However, this pattern must be interpreted with caution, as assessment density is low in the intermediate post-injury period (approximately 5–10 weeks), introducing uncertainty about the true underlying trajectory. During this interval, clinically relevant changes may occur that are not captured within the EMSCI assessment scheme and therefore cannot be modeled.

The acute-to-transition and pre-chronic-to-chronic thresholds were determined using an amplitude-based approach. The amplitude was calculated as the range from the minimum y-value of the curve to the mean y-value in the chronic stage of EMSCI (days 300–400). Sensitivity values of 2% and 5% defined the acute-to-transition threshold (amplitude exceeding 2% to 5%), while the chronic stage began when it reached 95% to 98% of its full amplitude.

The transition-to-pre-chronic threshold was determined by analyzing the curve’s rate of change (first derivative). The amplitude of the derivative was isolated, its minimum identified, and 10% of the amplitude was added to this minimum to establish a cutoff level. This point was projected back onto the original regression curve, and it was checked whether any preceding days had the same y-value. If matching values were found, those points defined a transition-to-pre-chronic window; if not, the single projected point served as the threshold.

The 10% threshold was chosen not to detect the exact peak (where the derivative is zero), but rather the onset of slowed improvement. A lower threshold (e.g., 0–5%) identifies points too deep into the inflection region, after substantial recovery deceleration has already occurred, while a broader threshold (e.g., 20%) shifts the transition too early, into the still-linear recovery phase. With a cutoff of 10%, we aim to provide a stable and interpretable marker of “nearly flat” slope behavior, capturing the beginning of the pre-chronic phase.

We acknowledge that these threshold values were initially selected heuristically rather than derived from an underlying biological model. To address this, we performed systematic sensitivity analyses in which the amplitude‑ and derivative‑based thresholds were varied across a broad range (acute: 1–20%, chronic: 80–99% of total recovery amplitude; pre‑chronic derivative threshold: 5–40% of the smoothed derivative amplitude). Please find the detailed description in the Supplementary Material in the chapter “Sensitivity Analysis of Phase Threshold Definitions” and supplementary Fig. 13. Consequently, these thresholds should be interpreted as data-driven definitions of recovery stages rather than fixed biological transitions.

### Latent class analysis

In addition to evaluating recovery trajectories based on clinical parameters such as AIS grade and NLI, our objective was to identify different subgroups of patients based on the shape of their recovery trajectories. To achieve this, we applied latent class mixed models (LCMM), which allow the identification of unobserved (latent) subpopulations within longitudinal data [33]. A random forest regression was used to identify the different stages of recovery, as described in Sect. "Identifying stages".

### Additional metrics

The procedures described in *2.2* were also applied to SCIM and hand motor strength (HMS), the latter in a subset of patients with cervical lesions [34]. We introduce this HMS measure here aiming to create a clinically meaningful indicator of hand motor capacity that reflects potential usefulness in activities of daily living. HMS was quantified by converting the ISNCSCI motor scores of C6, C8, and T1 into binary values. Specifically, scores ranging from 0 to 2 were coded as 0, indicating that the muscle strength at that segment was insufficient to perform basic activities of daily living involving the hand. In contrast, scores of 3 or higher were coded as 1, representing sufficient muscle strength to support at least rudimentary hand function. This binary approach allowed for a simplified yet clinically meaningful quantification of hand motor ability across the relevant spinal segments. To optimize neural network performance, the SCIM and HMS values were scaled between − 1 and 1 prior to curve fitting. The predicted values were then rescaled to their original range. The sample size for the SCIM trajectory analysis was 3622 patients and 11,645 data points, constrained by the availability of SCIM data. For the HMS path, 2548 patients and 8 832 data points with cervical injuries were included (the same inclusion criteria as above for both SCIM and HMS: first ISNCSCI assessment within 40 days and at least one additional assessment within 400 days after injury). The replication of the trajectory model across SCIM and HMS served as an internal sensitivity analysis, indicating that the identified phase boundaries are not specific to a single neurological scale but generalize to functional and hand‑specific outcomes.

## Results

A total of 4407 individuals with traumatic SCI were included in the analysis of the recovery trajectory using INCS. The mean age at the time of injury was 46.5 years (SD=18.8) and 78.9% of the cohort were male. On average, the initial ISNCSCI assessment was conducted 17.3 days (SD=11.6) after injury, and participants underwent a mean of 3.5 (SD=1.1) ISNCSCI assessments during the observation period. Cervical lesions represented the highest proportion of injuries (58.0%) and the AIS grades were distributed as follows: A (42.9%), B (12.2%), C (18.9%), and D (26.0%). An overview of the characteristics of the patients is provided in Table 1.

**Table 1:** Characteristics of the study population

Variable	Value
Age (years)	46.54 (18.77)
Sex (% male)	78.9%
Days until the Initial ISNCSCI assessment	17.32 (11.55)
Number of ISNCSCI assessments	3.45 (1.14)
*Initial AIS: n (%)*	
A	1891 (42.91%)
B	537 (12.19%)
C	833 (18.9%)
D	1146 (26%)
*Initial NLI: n (%)*	
Cervical (C1–C8)	2555 (57.98%)
Thoracic (T1–T10)	911 (20.67%)
Thoracolumbar (T11–L2)	941 (21.35%)

Metric values are shown as mean (SD), nominal and ordinal values are shown as absolute frequency (%)

Our study utilized three different modeling approaches (LOESS, random forests, and neural networks) to investigate the temporal dynamics of neurological recovery following SCI. Most models revealed a sigmoidal trajectory, beginning with an initial asymptotic phase that corresponds to the acute stage. During this initial acute phase (up to approximately 2 weeks post-injury), changes in neurological function were minimal.

This is followed by an increasing slope, marking the transition phase—characterized by the highest rate of spontaneous neurological change—which extends to approximately 9–10 weeks post-injury (see Fig. 3). In contrast to our initial assumption that the chronic stage would follow immediately after the transition phase, the analyses consistently identified an additional intermediate phase. This pre-chronic stage is characterized by ongoing but non-linear variations in recovery and extends up to approximately 7–8 months post-injury. Thereafter, the curve plateaued, signaling the entry into the chronic stage, where further neurological gains were minimal.

Figure 3 shows the overall recovery trajectory based on INCS, modeled using random forest regression, for the entire study population—encompassing all neurological levels of injury and AIS grades. Vertical red lines mark the data-driven thresholds that separate the stages of recovery. Measurement density in the acute stage is lower during the first few days post-injury; accordingly, the acute threshold (indicated by a dotted line) should be interpreted with caution due to increased uncertainty in this early period. Notably, the figure also highlights time intervals with sparse measurement density, particularly outside the predefined EMSCI assessment windows. These low-density regions, corresponding to the so-called "additional" EMSCI time frames, show greater fluctuation in the predicted curve (blue shaded area), indicating increased uncertainty in the estimated trajectory during these periods.

The thresholds separating the stages of recovery in the overall cohort were largely consistent across all modeling approaches (Table 2), with the characteristic four-phase pattern observed in random forest (Fig. 3), LOESS (supplementary Fig. 15a), and neural network (supplementary Fig. 15b) models.

**Table 2:** Values are reported in days after injury

Cohort	LOESS			Random forest		
	Acute	Pre-chronic	Chronic	Acute	Pre-chronic	Chronic
Overall	12–15	61–74	200–210	11–15	63–71	198–208
AIS A	3–7	119	245–276	12–16	68–75	221–323
AIS B	2–5	127	214–283	17–20	73	200–220
AIS C	14–18	74	241–321	13–18	70	225–241
AIS D	13–15	70–71	194–212	11–14	66–68	187–203
Cervical	11–14	70–71	209–218	11–15	67–71	202–213
Thoracic	1–4	120–121	300–328	14–17	61–71	208–209
Thoracolumbar	12–16	72–76	134–300	13–17	68–73	134–142

Cohort	Neural network			Merged^1^		
	Acute	Pre-chronic	Chronic	Acute	Pre-chronic	Chronic
Overall	12–16	55–61	242–288	12–15	60–69	213–235
AIS A	12–18	107	261–303	9–14	98–100	242–301
AIS B	11–16	152	231–301	10–14	117	215–268
AIS C	13–16	107	277–312	13–17	84	248–291
AIS D	10–15	52–59	210–269	11–15	63–66	197–242
Cervical	12–15	57–63	270–310	11–15	65–68	227–247
Thoracic	12–16	90	300–328	9–12	90–94	269–288
Thoracolumbar	10–14	90	221–295	12–16	77–80	163–246

Thresholds correspond to: acute (acute-to-transition), pre-chronic (transition-to-pre-chronic), and chronic (pre-chronic-to-chronic)

^1^Derived mean values from the respective cohort across all models

**Fig. 4:**
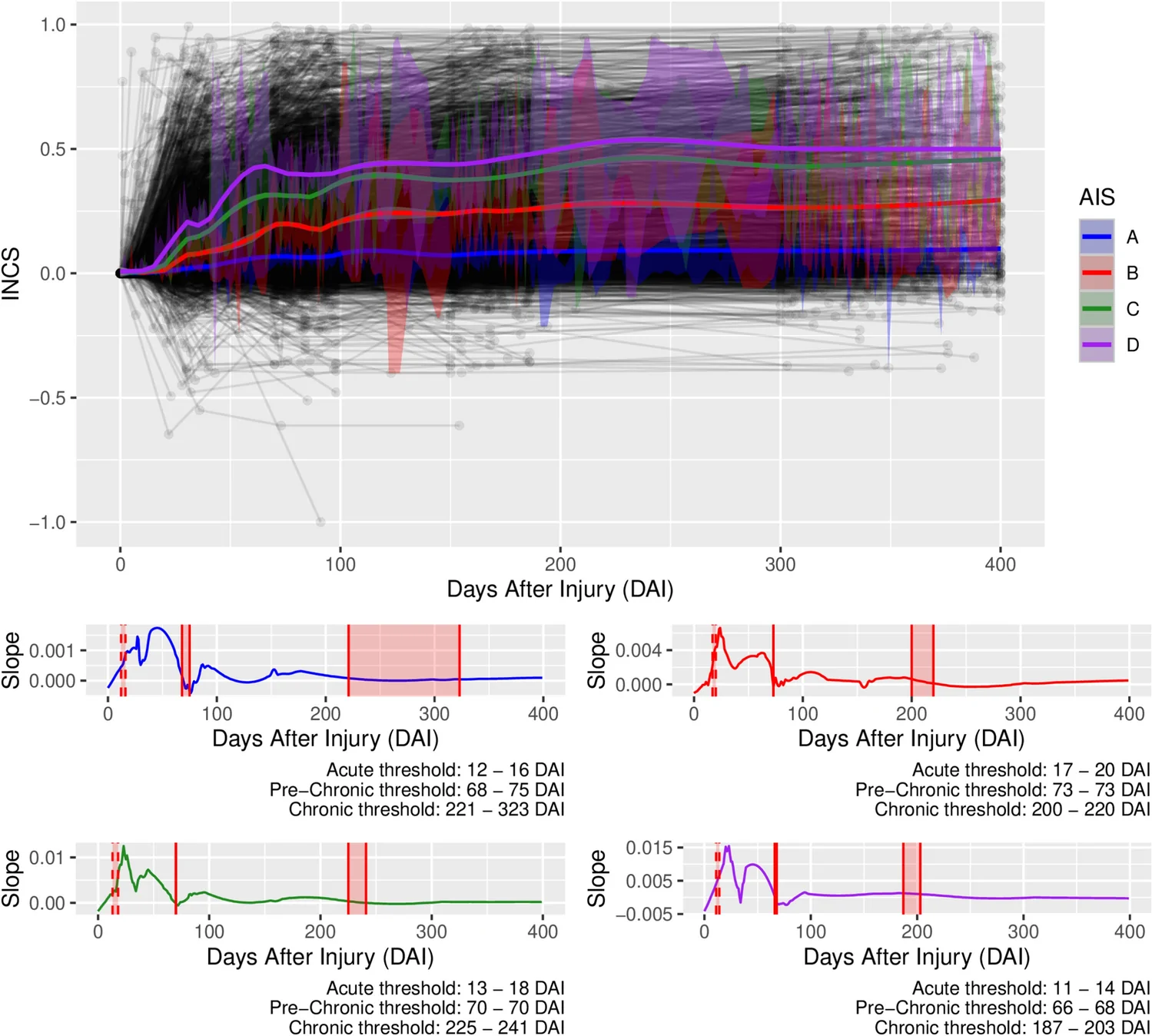
Neurological recovery trajectory based on INCS using random forest regression: scatterplot of all available INCS values plotted against days after injury, overlaid with the random forest regression curves separately for the AIS groups. Dots represent individual neurological assessments, with lines connecting them to illustrate individual patient trajectories. The upper panel shows the estimated recovery trajectories, while the lower panel displays the first derivative (slope) of the smoothed curves. Vertical lines (red) indicate the data-driven thresholds between recovery stages. *INCS* Integrated neurological change score, *DAI* days after injury, *AIS* American Spinal Injury Association Impairment Scale

**Fig. 5:**
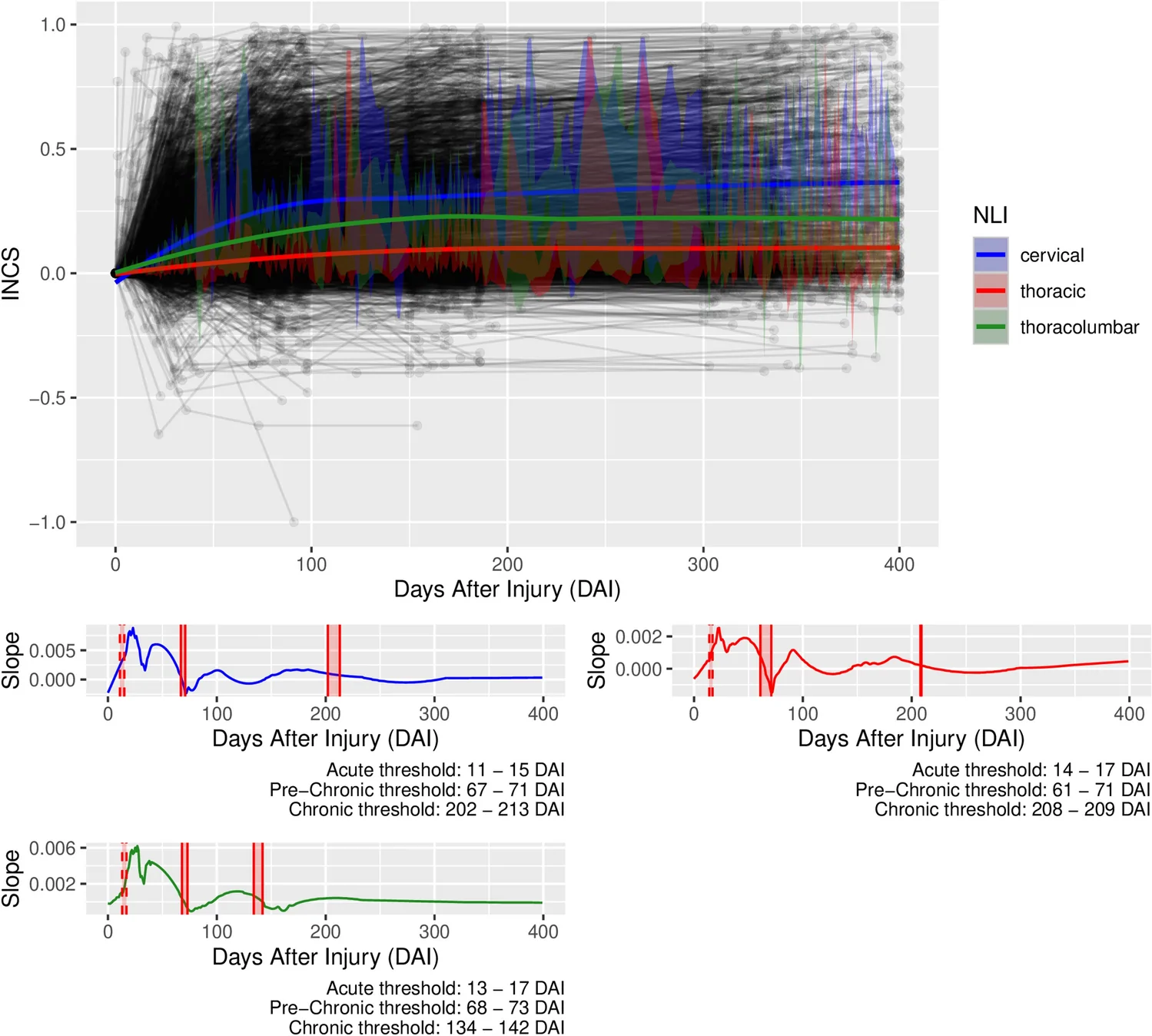
Neurological recovery trajectory based on INCS using random forest regression: scatterplot of all available INCS values plotted against days after injury, overlaid with the random forest regression curves separately for the NLI groups. Dots represent individual neurological assessments, with lines connecting them to illustrate individual patient trajectories. The upper panel shows the estimated recovery trajectories, while the lower panel displays the first derivative (slope) of the smoothed curves. Vertical lines (red) indicate the data-driven thresholds between recovery stages. *INCS* Integrated neurological change score, *DAI* days after injury, *NLI* neurological level of injury

While the overall temporal structure of recovery was largely preserved across subgroups, analyses revealed substantial differences in the magnitude of neurological improvement depending on injury severity and neurological level (Table 2, Figs. 4 and 5, supplementary Figs. 16–19).

Across AIS subgroups, the random forest model identified largely similar temporal patterns. However, among neurological levels, individuals with thoracolumbar injuries reached the chronic stage earlier than those with cervical or thoracic injuries (Figs. 4 and 5).

Deviations in the timing of recovery phases were primarily observed in the LOESS and neural network models, particularly among more severely injured individuals or those with thoracic-level injuries. Specifically, in the LOESS model, AIS A and B groups exhibited a very short acute phase, followed by a delayed onset of the pre-chronic phase compared to the overall population (supplementary Fig. 16). A similar pattern was evident in the thoracic group (supplementary Fig. 17).

In the neural network model, individuals with AIS A, B, and C injuries showed a generally delayed pre-chronic threshold, extending the transition phase beyond what was observed in the overall cohort (supplementary Fig. 18). Only the AIS D group aligned more closely with the reference timing. Comparable delays were observed in the thoracic and thoracolumbar groups, whereas the cervical subgroup followed a pattern more consistent with the overall trajectory (supplementary Fig. 19).

Regarding the magnitude of recovery (*y*-axis: INCS), substantial and consistent differences were observed across all modeling approaches. Individuals with cervical injuries demonstrated the greatest neurological improvement, followed by those with thoracolumbar injuries, while thoracic injuries were associated with the lowest recovery potential (Fig. 5, supplementary Figs. 17 and 19). A similar pattern was evident across AIS grades: those with AIS D injuries exhibited the most pronounced recovery gains, followed by AIS C and B. Injuries classified as AIS A were associated with only minimal neurological improvement across the observation period—consistent with the limited recovery potential in this subgroup (Fig. 4, supplementary Figs. 16 and 18).

### Alternative metrics

To assess whether the observed temporal pattern of neurological recovery is also reflected in functional improvements, we analyzed the recovery trajectories of the SCIM and HMS, a neurological measure for hand motor function, alongside the INCS-based models. These additional metrics were selected to evaluate whether the identified time frames align between neurological change in the ISNCSCI assessments and clinically relevant functional outcomes.

**Fig. 6:**
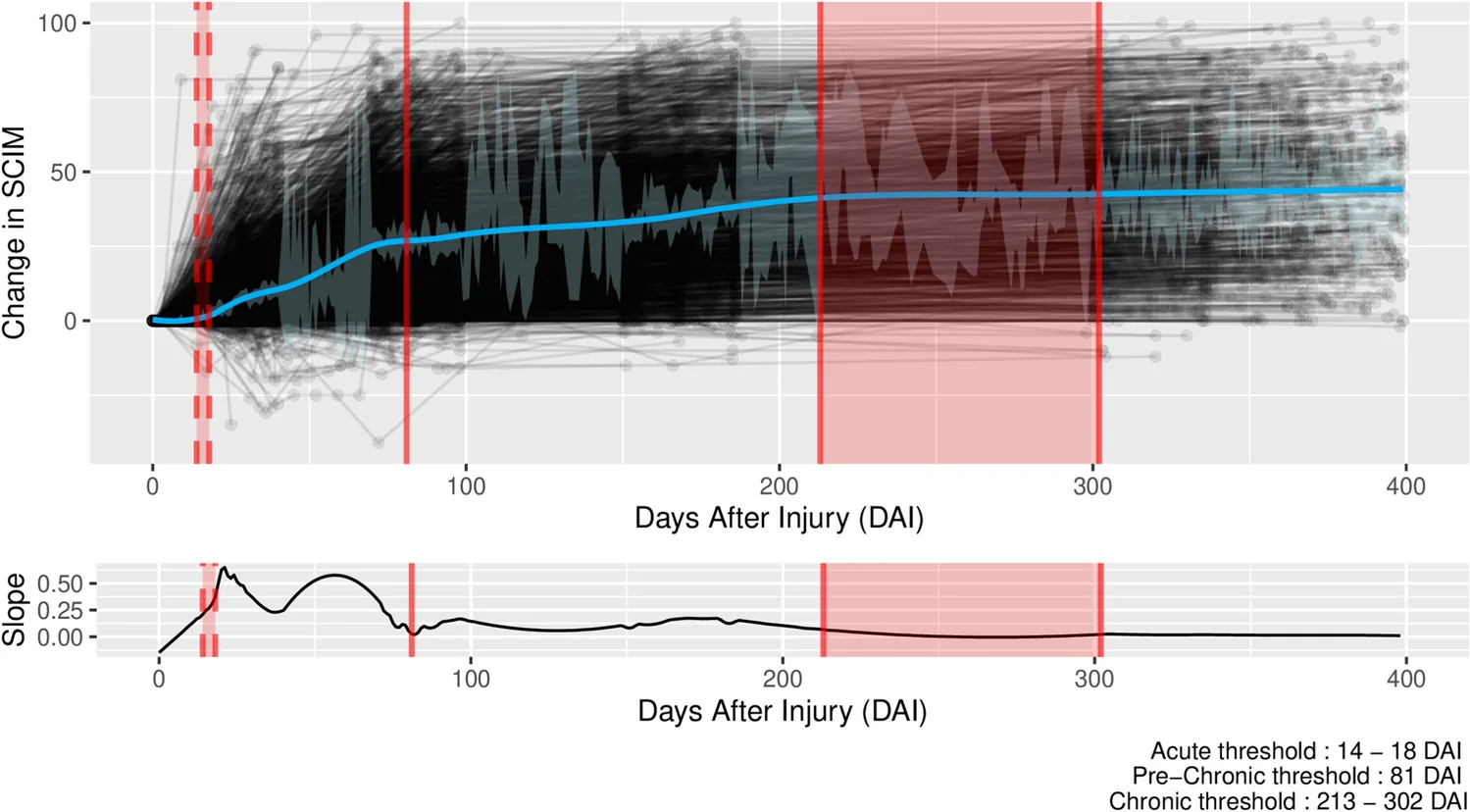
Functional recovery trajectory based on change in the total SCIM using random forest regression: scatterplot of all available SCIM assessments plotted against days after injury, overlaid with the random forest regression curve (blue line). Dots represent individual functional assessments, with lines connecting them to illustrate individual patient trajectories. The upper panel shows the estimated functional recovery trajectory, while the lower panel displays the first derivative (slope) of the smoothed curve, reflecting the rate of functional change over time. Vertical lines (red) indicate the data-driven thresholds between recovery stages. *SCIM* Spinal cord independence measure, *DAI* days after injury

As shown in Fig. 6, Table 3*,* and supplementary Fig. 20, the transition phase—during which the most substantial functional gains were observed—extended further when assessed using SCIM compared to INCS. In the SCIM-based trajectories, this phase lasted up to approximately 12 weeks post-injury in the random forest and LOESS models, and even to 17 weeks in the neural network model. While the duration of the transition phase differed, the thresholds marking the onset of the transition phase (i.e., the acute threshold), as well as the chronic phase remained largely consistent with those identified in the INCS trajectories.

**Table 3:** Values in days after injury (DAI)

Method	SCIM			HMS		
	Acute	Pre-chronic	Chronic	Acute	Pre-chronic	Chronic
Random forest	14–18	81	213–302	11–15	74	327–345
LOESS	15–18	85	219–320	12–15	71	322–343
Neural network	15–18	81	233–275	11–15	120	299–331
Merged^1^	15–18	82	222–299	11–15	88	316–340

^1^Derived mean values across all models

**Fig. 7:**
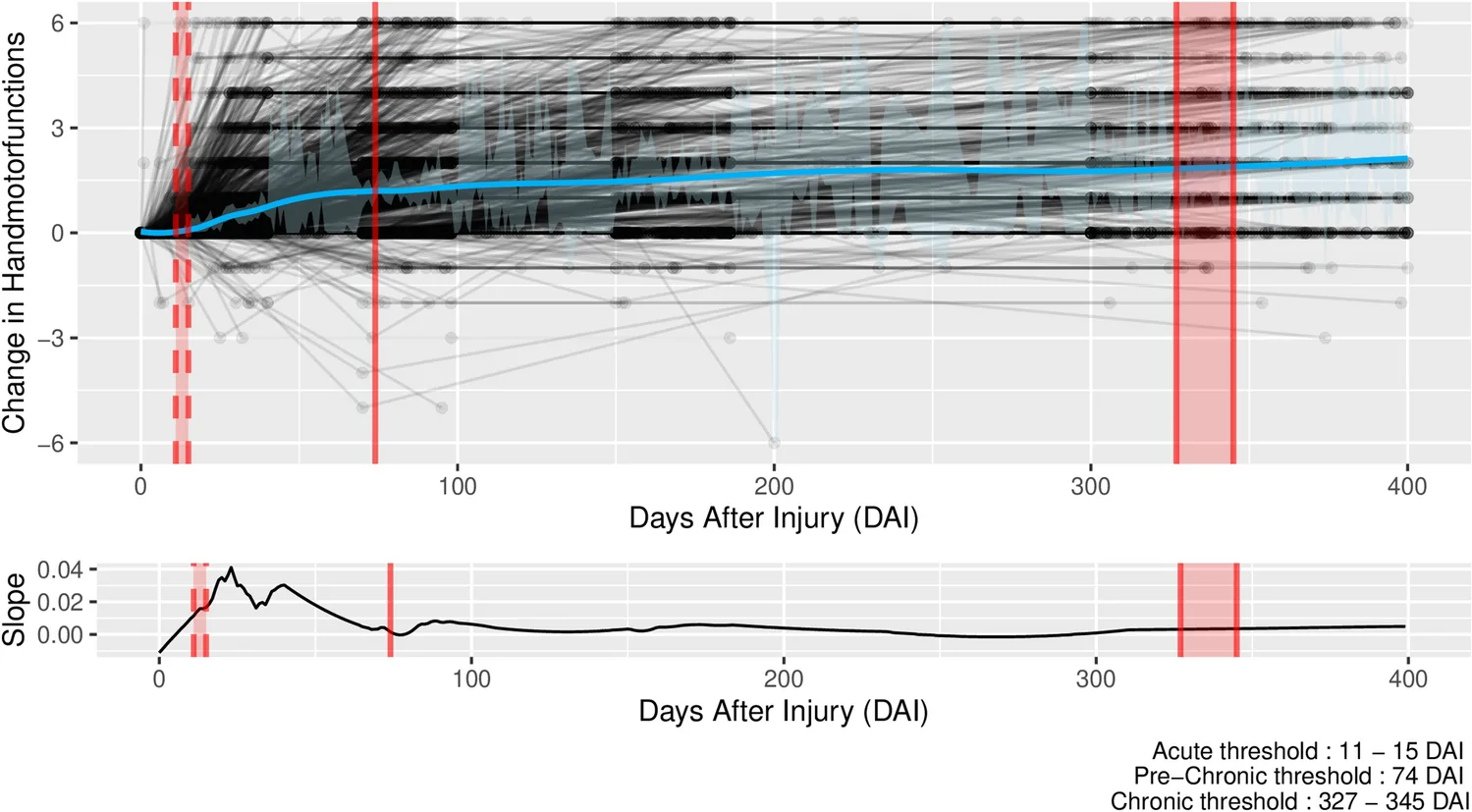
Neurological recovery trajectory based on changes in hand motor strength using random forest regression: scatterplot of all available HMS assessments (within the ISNCSCI assessment) plotted against days after injury, overlaid with the random forest regression curve (blue line). Dots represent individual assessments, with lines connecting them to illustrate individual patient trajectories. The upper panel shows the estimated recovery trajectory, while the lower panel displays the first derivative (slope) of the smoothed curve, reflecting the rate of change over time. Vertical lines (red) indicate the data-driven thresholds between recovery stages. *HMS* hand motor strength, *ISNCSCI* International standards for neurological classification of spinal cord injury

For the HMS analysis, we intentionally focused on upper extremity motor scores at myotomes C6, C8, and T1 to derive a targeted neurological measure for hand motor function. As shown in Fig. 7, Table 3, and supplementary Fig. 21, the HMS-based trajectory exhibited a transition phase of intermediate duration compared to those identified in the SCIM and INCS trajectories, extending to approximately 10–11 weeks post-injury in both the random forest and LOESS models. The neural network model again diverged slightly, with the transition phase lasting up to 17 weeks. Notably, the acute threshold was highly consistent across all metrics and modeling approaches (roughly 2 weeks). In contrast, the onset of the chronic phase occurred later in the HMS trajectory compared to INCS and SCIM, indicating a more prolonged period of functional recovery. The thresholds delineating the chronic phase also spanned broader time intervals in both SCIM and HMS analyses, which likely reflects the smaller sample sizes available for these outcome measures (SCIM: *n*=3622; HMS: *n*=2548). For SCIM and HMS, only thresholds from the overall cohort are reported in Table 3 due to these reduced sample sizes.

**Table 4:** Distribution of patient characteristics, AIS grades, and NLI across four latent classes

	Class 1	Class 2	Class 3	Class 4	*p*-value
Patient count	310	773	1135	2189	
Age (years)	49.65 (18.44)	48.73 (18.16)	49.63 (18.79)	43.73 (18.62)	<0.001
Sex (% male)	28 (74.84%)	595 (76.97%)	886 (78.06%)	1764 (80.58%)	0.029
Initial assessment	8.41 (6.56)	14.19 (9.2)	18.44 (11.45)	19.1 (12.09)	<0.001
Number of assessments	3.48 (1.27)	3.46 (1.18)	3.45 (1.11)	3.43 (1.13)	0.971
Initial UEMS^1^	28.5 (24)	30 (29)	33 (34)	50 (33)	<0.001
Initial LEMS^2^	32 (23)	26 (31)	13 (32.5)	0 (0)	<0.001
Chronic INCS^3^	0.77 (0.19)	0.63 (0.14)	0.35 (0.12)	0.04 (0.09)	<0.001
*Initial AIS*					<0.001
A	13 (4.19%)	52 (6.73%)	281 (24.76%)	1545 (70.58%)	
B	14 (4.52%)	92 (11.9%)	161 (14.19%)	270 (12.33%)	
C	84 (27.1%)	235 (30.4%)	318 (28.02%)	196 (8.95%)	
D	199 (64.19%)	394 (50.97%)	375 (33.04%)	178 (8.13%)	
*Initial NLI*					<0.001
Cervical	250 (80.65%)	591 (76.46%)	754 (66.43%)	960 (43.86%)	
Thoracic	10 (3.23%)	47 (6.08%)	120 (10.57%)	734 (33.53%)	
Thoracolumbar	50 (16.13%)	135 (17.46%)	261 (23%)	495 (22.61%)	

INCS in chronic phase (as determined in Table 2, merged overall), Nominal values and AIS grades in number of patients (percentage), metric values in mean (standard deviation), ordinal values (UEMS and LEMS) in median (interquartile range)

Classes ranked by the mean slope/recovery within each group

*P*-values are calculated using Kruskal–Wallis test for ordinal/metric values and Chi-squared- test for nominal values

^1^Upper extremity score

^2^Lower extremity score

### Latent classes

**Fig. 8:**
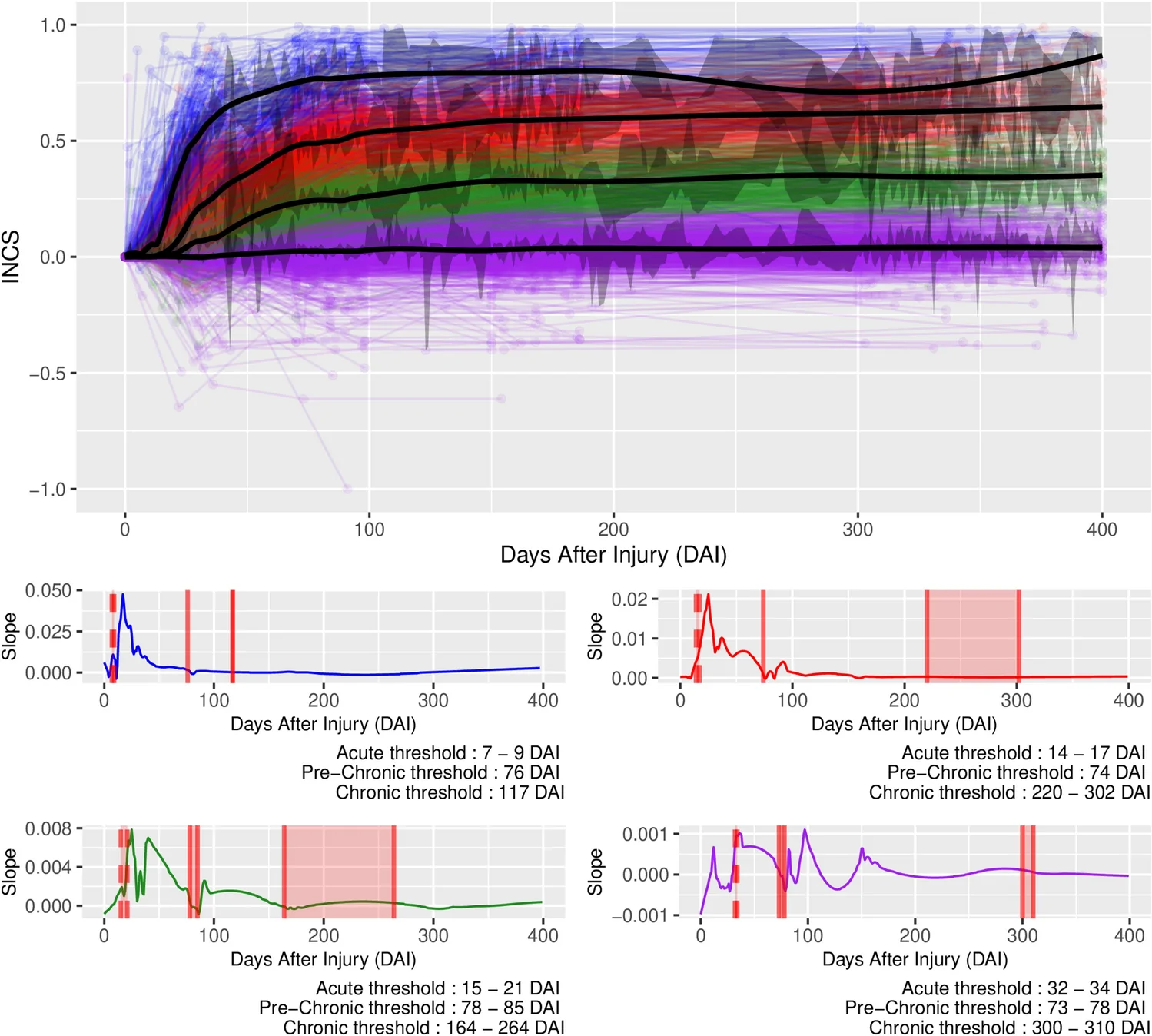
Latent class trajectories of neurological recovery based on the INCS: The top panel displays individual patient trajectories color coded by latent class, overlaid with the class-specific mean recovery trajectories derived from random forest regression (black lines). The lower panels show the respective first derivatives (slopes) and data-driven thresholds (vertical red lines) for the acute, transition, and chronic phases within each class. Class 1 shows the steepest and earliest transition, while class 4 remains largely flat throughout, indicating no recovery. Classes 2 and 3 follow intermediate courses. *INCS* Integrated neurological change score, *DAI* days after injury

To investigate whether distinct recovery profiles could be identified independent of traditional clinical classifications, we applied LCMM to the INCS data. This approach revealed four latent recovery classes, each exhibiting a distinct trajectory of neurological change over time (Table 4and Fig. 8).

Class 1 comprised individuals with the greatest overall neurological recovery, characterized by a steep transition phase and early attainment of the chronic stage. This group predominantly included patients with incomplete injuries (AIS C and D) and cervical, as well as thoracolumbar lesion levels. Class 4, in contrast, showed virtually no neurological recovery over the observation period. It consisted primarily of individuals with complete injuries (AIS A and B) and the highest proportion of thoracic lesions compared to the other classes. Classes 2 and 3 represented intermediate recovery profiles, with class 2 achieving slightly better and earlier recovery than class 3. While AIS grade and NLI were broadly predictive of class membership, they did not fully account for the observed recovery variability. Notably, some individuals with motor complete injuries (AIS A or B) achieved substantial recovery (classified in classes 1 or 2), while others with initially favorable clinical profiles (AIS C or D) demonstrated limited or no recovery (class 4). Additionally, recovery onset occurred slightly earlier in the better-recovering classes, suggesting that early neurological gains may be predictive of favorable long-term outcomes.

Pairwise comparisons showed significant age differences between classes 1, 2, and 3 versus class 4 (p<0.001). No pairwise differences in sex distribution were observed. Time from incident to initial INCS assessment differed across all classes except between Classes 3 and 4 (p<0.001 for all others). The INCS in the chronic phase (its beginning defined as in Table 2 for the overall cohort using the merged values) is significantly different between Classes 1 and 2 (p<0.05) and highly significant for all other (p<0.001). Initial AIS grade differed significantly across all comparisons (p<0.01 for Classes 1 vs. 2; p<0.001 otherwise), while initial NLI differed across all except Classes 1 and 2 (p<0.001 for others). While LEMS differed significantly between all class comparisons (p<0.001), UEMS showed significant differences only between Classes 1 and 3 (p<0.01) and 3 and 4 (p<0.01).

Building on the preceding analyses, it was evaluated whether the distinct subgroups exhibit significant changes beyond our proposed chronic threshold (approximately 7–8 months post-SCI). For this, only patients who had an assessment between 5–7 months and one after 360 days were considered. Each of the four latent classes show negligible late change (mean INCS spanning 0.0114–0.0366) with no statistically significant differences between classes (p=0.243). Class-specific means (SD): class 1, 0.023 (0.185); class 2, 0.037 (0.169); class 3, 0.036 (0.099); and class 4, 0.011 (0.057). Significant changes were observed within Classes 3 and 4 (p<0.001 and p=0.03, respectively). However, examination of the magnitude of these changes shows that they are very small. The median change in class 3 was 0.023 (IQR: 0.067), and in class 4 it was 0.007 (IQR: 0.042). Although the statistical tests indicate significance, the actual size of the changes is minimal, suggesting limited practical or clinical relevance.

## Discussion

Our findings demonstrate a consistent temporal structure of post-injury recovery in individuals with traumatic SCI across different modeling approaches (random forest, LOESS, neural networks), recovery metrics (INCS, SCIM, HMS), and patient subgroups. In particular, the transition phase, characterized by the most substantial neurological and functional gains, was consistently identified to occur roughly within the first 10 weeks after injury. The timing of this critical window of improvement remained remarkably stable across our analyses. Beyond the transition phase, further recovery was typically limited to slower and more variable gains. Consistent with this plateau, when restricted to an observation period between 5–7 months and after 360 days, no meaningful late change across or within classes are observed, further validating the chronic threshold. While neurological and functional improvements may still occur beyond 10 weeks, the majority of measurable change appears to take place within this early post-injury period. These findings provide a temporal map of spontaneous recovery, supporting the concept of a defined window of increased neuroplastic potential. While our observational data do not test intervention efficacy, they identify a critical period of rapid change that likely represents an important time frame for therapeutic interventions.

While the overall timing of recovery stages proved robust across methods and metrics, particular caution is warranted when interpreting the end of the acute phase — and thus the onset of the transition phase. In individuals with more severe injuries, early ISNCSCI assessments are often delayed due to clinical factors such as sedation, intubation, or impaired consciousness. In our analysis, individuals with poorer recovery outcomes (e.g., latent class 4) underwent their initial neurological assessment significantly later than those with better recovery trajectories (Table 2). This suggests that the seemingly prolonged acute phase in more severely injured patients may be influenced by clinical feasibility rather than pathophysiological injury progression.

When examining recovery trajectories based on SCIM and HMS, we observed that they tended to plateau later than those derived from INCS. This delay may, in part, be attributed to training effects that influence functional performance over time and thereby potentially skew the depiction of true neurological recovery. Unlike the INCS, which captures change in neurological function more directly, SCIM and HMS reflect performance-based outcomes that are subject to rehabilitation intensity, compensatory strategies, and environmental factors. Recent literature also indicates that the recovery pattern may vary depending on the proximity to the spinal cord: Balbinot et al. demonstrated that distal muscles of the upper limb show limited and delayed motor recovery compared to proximal muscles [34]. Brüningk and colleagues similarly emphasized that aggregated sum scores such as upper extremity motor scores (UEMS) and lower extremity motor scores (LEMS) may oversimplify recovery patterns and advocated for data-driven methods that reflect myotome-specific motor recovery dynamics [35].

Although SCI classification primarily relies on the NLI and AIS grade, our latent class analysis revealed additional stratification patterns that are not fully captured by these conventional metrics. Generally, LCMM has become an increasingly valuable tool in SCI, as well as traumatic brain injury (TBI) research, enabling the identification of unobserved subgroups and modeling heterogeneity in recovery trajectories and outcomes [36–38]. In our analysis, a four-class solution was selected to align with prior work by Badhiwala et al., who applied group-based trajectory modeling to longitudinal upper extremity motor scores in a large cohort of individuals with cervical incomplete SCI. Their study identified four clinically meaningful trajectories over 1 year, demonstrating the utility of this approach for uncovering data-driven recovery profiles [37]. Notably, in our study, the distinction between recovery profiles in Classes 2 and 3 was primarily driven by improvements in lower extremity function — a dimension not adequately differentiated by overall motor scores or AIS grade. This observation supports the potential value of more targeted assessments, such as lower-extremity-focused evaluations, which could streamline follow-up, minimize missing data, and enhance sensitivity to meaningful functional gains within specific patient subgroups.

### Limitations

The present study utilizes multiple regression models to characterize the underlying trend in neurological recovery following SCI. However, the study is limited by gaps in data availability. Specifically, the sparse measurement density in certain post-injury time intervals—particularly those outside the predefined EMSCI assessment windows, and the very acute phase (first days after injury)—introduces an uneven distribution of assessments along the post-SCI time interval, leading to greater variability and uncertainty in predicted recovery curves. For SCIM trajectories in particular, early-stage changes may be overestimated, as patients in the acute phase are typically highly dependent on caregivers and therefore present SCIM scores that cluster near zero, regardless of underlying neurological function. Additionally, the applied modeling approaches do not explicitly account for within-subject correlations arising from repeated assessments. However, the primary aim of the study was to estimate a population-level recovery trajectory rather than subject-specific patterns of change. Furthermore, most participants contributed only a limited number of assessments, typically two observations, restricting the ability to reliably estimate individual non-linear trajectories.

Second, the reconstructed trajectory relies on combining observations from individuals assessed at different times after injury. Consequently, the estimated curve should be interpreted as a population-average representation of recovery rather than a direct longitudinal characterization of individual patients. Although mixed-effects approaches could formally model within-subject dependence, the sparse repeated-measures structure of the present dataset provides limited information for estimation of complex subject-specific recovery patterns.

Finally, the identified plateau should be interpreted as an estimate of the point at which recovery stabilizes at the population level. Individual patients may exhibit substantial variability around this average trajectory.

The precision and generalizability of our neurological trend analysis are consequently constrained by these inherent data availability limitations. Our findings underscore the importance of assessment frequency for the stability and interpretability of model-based estimates, as denser sampling would likely yield more precise and reliable trajectory estimates.

## Conclusion

The trajectory of neurological recovery after SCI comprises four distinct stages: an acute phase (up to 2 weeks post-injury) with minimal change, a transition phase (∼ 2 to 9–10 weeks) marked by substantial neurological improvement, a pre-chronic phase (∼ 10 weeks to ∼ 7–8 months) characterized by sustained yet diminishing improvement, and a chronic phase (beyond ∼ 7–8 months) in which recovery plateaus. Across all models and outcome measures, neurological change consistently preceded functional improvements captured by SCIM and hand motor strength, supporting the notion that functional recovery is influenced by, but temporally lags behind, neurological restoration.

These findings provide a data-driven framework for staging SCI recovery and show that meaningful neurological gains occur predominantly within the first 10 weeks post-injury. In clinical practice and trial design, assessment schedules and intervention timing should be adapted accordingly: obtain a high-quality ISNCSCI baseline as early as feasible; consider the potential for higher impact of neurorestorative and rehabilitation efforts within the period of rapid recovery (~ 10 weeks); and include a confirmation time point at 7–8 months to document entry into the chronic stage. In light of the observed variability in lower extremity recovery across latent classes, we further recommend incorporating lower-extremity-focused assessments to improve sensitivity to clinically meaningful change.

## Supplementary Information

Below is the link to the electronic supplementary material.

Supplementary file1 (.pdf 3926 KB)

## Data Availability

The data used in this study were obtained from the European Multicenter Study about Spinal Cord Injury (EMSCI) under a data-use agreement (Data Providing Agreement: EMSCI_V14_OF_Murnau_20230116_HNP; signed on Feb 8th 2023). The corresponding author does not have permission to redistribute the data. Access requests should be directed to the EMSCI study group (https://www.emsci.org).

## Declarations

### Generative AI and AI-assisted technologies

The authors acknowledge the use of large language models (ChaptGPT) to support the drafting of text within this article. Any suggestions obtained were critically reviewed and rephrased where needed. The authors emphasize that support was limited to grammatical and editorial phrasing. The authors take full responsibility for the content of the publication.
