# Supplementary material for: A data-driven approach to define the stages and patterns of neurological recovery after traumatic spinal cord injury
Source: J Neurol. 2026 Aug 19;273(9):531. doi: 10.1007/s00415-026-14065-9 (PMC13490207; doi:10.1007/s00415-026-14065-9)
Supplement: Supplementary file 1 [file 415_2026_14065_MOESM1_ESM.docx]

# Supplementary Material: Extended Methods

We first extracted ISNCSCI and SCIM scores including their sub-scores from the EMSCI dataset and calculated the INCS as a metric for neurological change. To examine the trajectory of neurological change, we mapped INCS values against days after injury (DAI) resulting in a scatterplot of all assessments (n = 17932) and individual patient trajectories (n = 5102) as depicted in supplementary figure 9. Given the observable heterogeneity of SCI recovery, the study’s aim was to articulate a generalized recovery trajectory for traumatic SCI.

Supplementary figure 9 Scatterplot of assessments over time
DAI: days after injury

## Data Cleaning

The density of assessments is unequally distributed along the post-SCI time interval, presenting regions of high assessment density, corresponding to the EMSCI’s examination periods, interspersed with sparse intervals in the so called "additional" time frames (figure 1 and supplementary figure 9). Also, we observed a definite decline in the density of assessments in the chronic stage. To mitigate bias in our trend evaluation due to a decline in measurement density, we analyzed the temporal distribution of assessment density, identifying the DAI value at which this density drops below a threshold of 5% of the amplitude of the density plot within an observation period of 1 week (supplementary figure 10 part (a)).All assessments conducted beyond 400 days post-SCI were removed from the dataset.

Evaluating neurological function over time requires an initial measurement which serves as a reference point. Ideally, this assessment would take place upon hospital admission. However, a substantial portion of our patient sample underwent their initial ISNCSCI assessment either during or after the "acute I" phase of EMSCI (days 16-40). Additionally, beyond day 41, the density of first assessments declines to below 5% of the amplitude within a one-week observation period. Therefore we excluded patients whose initial ISNCSCI has been obtained beyond 40 days post-injury (supplementary figure 10 part (b)).

Supplementary figure 10 Assessment density and density-based time cutoff for (a) last assessment and (b) first assessment.

We chose not to remove outliers from the dataset, as the data represent clinical observations and thus reflect real-world clinical variability. Excluding patients with exceptionally good or poor outcomes would fail to account for the full spectrum of recovery patterns observed in practice. However, we observed that more than 10% of the remaining sample exhibited substantial changes in their NLI during the observation period (i.e., shifts of more than three levels) which appeared implausible in the context of typical recovery patterns. To address this, we applied an additional filter: for each initial NLI level (C1–C8, T1–T12, L1-L2), we identified and excluded the top 5% of patients with the most pronounced upward shifts and the bottom 5% with the most pronounced downward shifts in NLI change, relative to others with the same initial level. This leaves a total of 4407 patients and 15191 data points (i.e., ISNCSCI assessments). The remaining assessments and the trajectories over time per patient can be seen in supplementary figure 11 (a) and (b) respectively.

|    (a) Scatterplot of assessments over time |  |    (b) Scatterplot of assessments over time with individual patient trajectories |
| --- | --- | --- |

Supplementary figure 11 Neurological assessments over time of the filtered dataset
DAI: Days after incident

## Regression Analyses

Several methodologies were employed for the regression analysis including estimated scatterplot smoothing (LOESS) [26–29], random forests [30], and neural networks [31] for machine learning-based predictions. Each algorithm and its associated regression curves were tuned using a hyper-grid of selected hyperparameter [32]. For LOESS, we tested span values from 0.2 to 0.65 in 0.5 increments. For the random forest, we tuned the number of trees (ranging from 5 to 200 in steps of 5) and mtry values (from 1 to 5 in steps of 1).For the neural network model, several hyperparameter were tuned. Decay values were explored from 0.0025 to 0.1 in 11 steps, the number of units in the hidden layer from 5 to 20 in steps of 1, and the maximum number of iterations from 100 to 1000 in steps of 100. For each regression method, the hyperparameter that minimized RMSE were selected. To mitigate the risk of overfitting, a combination of five-fold cross-validation and an early stopping mechanism were incorporated [39]. Early stopping was defined by consecutive improvements in RMSE plateauing or declining over several successive intervals. To reduce fluctuations in the Random Forest models, a one-pass loess smoothing was applied upon their predictions, optimizing its span based on RMSE. For each regression method, 95% confidence bands were computed. For the random forest, bootstrap percentiles were used. For LOESS and the neural network, analytical confidence intervals were applied; however, for the neural network, bootstrapping was employed to estimate the standard error. All bootstrapping procedures were based on 1,000 resamples.

Acknowledging that the overall trajectory of neurological change might differ substantially among subgroups of our study population based on the neurological level, as well as severity of SCI, we split the overall dataset based on clinical parameters such as the AIS grades (A - D) and the NLI (cervical, C1 - C8; thoracic, T1 - T10; thoracolumbar, T11 - L2). The selection of hyperparameter process was repeated for and upon each of these cohorts. The statistical metrics and final parameters for each model and cohort can be found in table 5.

|  | LOESS | | Random Forest | | Neural Network | | | |
| --- | --- | --- | --- | --- | --- | --- | --- | --- |
|  | span | RMSE | trees | RMSE | units | decay${}^{1}$ | iterations${}^{2}$ | RMSE |
| Overall | 0.2 | 0.22 | 50 | 0.22 | 20 | 0.0025 | 500 | 0.22 |
| AIS A | 0.6 | 0.12 | 35 | 0.12 | 14 | 0.0025 | 100 | 0.12 |
| AIS B | 0.65 | 0.19 | 40 | 0.20 | 20 | 0.01225 | 900 | 0.19 |
| AIS C | 0.25 | 0.21 | 20 | 0.23 | 7 | 0.0025 | 100 | 0.22 |
| AIS D | 0.2 | 0.22 | 40 | 0.24 | 8 | 0.0025 | 900 | 0.22 |
| cervical | 0.25 | 0.23 | 50 | 0.23 | 18 | 0.0025 | 800 | 0.23 |
| thoracic | 0.65 | 0.14 | 20 | 0.15 | 19 | 0.0025 | 100 | 0.14 |
| thoracolumbar | 0.3 | 0.19 | 50 | 0.21 | 18 | 0.022 | 800 | 0.20 |

Table 5 Final hyperparameter and statistical metrics for INCS.

^1^weight decay
^2^maximum number of iterations

The Regression analyses yielded sigmoidal-shaped curves. Instead of an immediate plateau after the steepest increase (neurological window of opportunity), a phase of slight variations persisted for weeks before stabilizing in the chronic stage. Thus, the recovery trajectory comprises four stages: the acute phase, a transition phase with significant neurological change, a pre-chronic phase with non-linear variations, and the chronic phase where it plateaus. An example of a regression curve using the random forest method is shown in supplementary figure 12.

Supplementary figure 12 Generalized curve of neurological recovery using a random forest regression model
Abbreviations: INCS, Integrated Neurological Change Score; DAI, days after injury

The acute to transition and pre-chronic to chronic thresholds were determined using an amplitude-based approach. Amplitude was defined as the range from the curve’s minimum y-value (INCS) to the mean y-value in the EMSCI chronic stage (days 300–400). Sensitivity values of 2% and 5% defined the acute to transition threshold (amplitude exceeding 2% to 5%), while the chronic stage began when it reached 95% to 98% of its full amplitude.

The transition to pre-chronic threshold was identified using the curve’s rate of change (first derivative). The derivative’s amplitude was isolated and smoothed using one-pass LOESS with an RMSE-optimized span. A 10% threshold of the smoothed amplitude defined a range of days below this value, selecting the earliest one. This point was mapped back to the regression curve to check for preceding equal y-values; if found, a pre-chronic threshold window was set; otherwise, the single DAI value defined the threshold. The thresholds derived from the random forest regression curve are shown in Figure 3.

**Sensitivity Analysis of Phase Threshold Definitions**

Because the phase boundaries were defined using amplitude- and derivative-based thresholds, additional analyses were performed to evaluate the impact of alternative parameter choices.

For the acute phase, thresholds ranging from 1–20% of the total recovery amplitude were examined. Lower thresholds identified the onset of recovery earlier, whereas higher thresholds delayed the estimated transition by requiring a greater proportion of the overall recovery to occur before the threshold was reached (supplementary figure 13A). The selected range of 2–5% was chosen to capture the earliest sustained increase in the population recovery trajectory while avoiding excessive sensitivity to minor fluctuations.

For the chronic phase, thresholds between 80–99% of the total recovery amplitude were evaluated. As expected, lower percentages resulted in earlier classification of the chronic phase, while higher percentages delayed the threshold until recovery approached its maximal observed level (supplementary figure 13C). The chosen range of 95–98% was intended to identify the plateau region where additional recovery was minimal.

The pre-chronic threshold was determined from the first derivative of the recovery curve. To assess the influence of this parameter, thresholds corresponding to 5%, 10%, 30% and 40% of the smoothed derivative amplitude were considered. Lower thresholds produced broader pre-chronic earlier transition estimates, whereas higher thresholds resulted in narrower intervals and later transition estimates. Among the evaluated values, 10% provided a balance between premature classification during ongoing recovery and delayed classification after recovery had substantially decelerated (supplementary figure 13b).

Supplementary figure 13 Classification of thresholds depending on used sensitivity based on the overall random forest model. Panel a) shows the start of the acute threshold for sensitivity values between 1-20%, b) shows the windows for the pre-chronic phase for thresholds of 5%, 10% and 20% of the slopes amplitude and c) shows the start of the chronic phase for sensitivities between 1-20%, i.e., 80-99% of the curves amplitude.

These analyses demonstrate that the estimated phase boundaries depend on the selected operational definitions, particularly for the transition and chronic phases. Consequently, the identified thresholds should be interpreted as data-driven approximations of clinically meaningful recovery stages rather than fixed biological boundaries.

The above-mentioned procedures, encompassing the data filtering and regression exercises, were also performed on the SCIM and a rating for hand motor strength (HMS), the latter only in a subset of the dataset consisting of patients who sustained cervical lesions [34]. HMS was quantified by converting ISNCSCI motor scores of C6, C8, and T1 into binary values: scores of $0-2$ were assigned 0 (insufficient for daily activities), while scores $\geq$ 3 were assigned 1 (indicating potential for basic hand function). To optimize neural network performance, SCIM and HMS values were scaled between -1 and 1 before curve calculation. Predicted values were then rescaled to their original range. The statistical metrics and final parameters for each model regarding the SCIM and HMS metrics can be found in table 6 .

|  | LOESS | | Random Forest | | Neural Network | | | |
| --- | --- | --- | --- | --- | --- | --- | --- | --- |
|  | span | RMSE | trees | RMSE | units | decay${}^{1}$ | iterations${}^{2}$ | RMSE |
| SCIM | 0.2 | 0.18 | 25 | 0.19 | 20 | 0.0025 | 400 | 0.18 |
| HMS | 0.3 | 0.25 | 30 | 0.25 | 20 | 0.0025 | 100 | 0.25 |

Table 6 Final hyperparameter and statistical metrics for SCIM and HMS.

RMSE was scaled to values between -1 and 1 for better comparability
^1^weight decay
^2^maximum number of iterations

## Latent Class Analysis

In addition to evaluating recovery trajectories based on clinical parameters such as AIS grade and NLI, we aimed to identify distinct subgroups of patients based on the shape of their recovery trajectories. To achieve this, we applied latent class mixed models (LCMM), which allow for the identification of unobserved (latent) subpopulations within longitudinal data [33]. A Random Forest regression was used for identifying the different stages of recovery.

# Additional Results

## Latent Class Analysis

Four classes were identified as the most appropriate solution, based on both the relative group sizes and the meaningful differences observed between groups (see 3.2). A five-class solution introduces an additional, very small group of individuals who showed neurological decline over time (n = 35; 0.79% of the sample), alongside one class of non-recoverers, two classes of average recoverers, and one class of good recoverers (see supplementary figure 14). Patient characteristics as well as initial AIS grades and NLI can be seen in table 7.

|  | **Class 1** | **Class 2** | **Class 3** | **Class 4** | **Class 5** | p-value |
| --- | --- | --- | --- | --- | --- | --- |
| Patient Count | 311 | 767 | 1108 | 2186 | 35 |  |
| Age (years) | 49.71 (18.44) | 48.75 (18.20) | 49.72 (18.74) | 43.64 (18.53) | 51.26 (22.57) | $<0.001$ |
| Sex (% male) | 233 (74.92%) | 590 (76.92%) | 864 (77.98%) | 1762 (80.60) | 28 (80.00%) | $0.057$ |
|  |  |  |  |  |  |  |
| Initial Assessment | 8.44 (6.57) | 14.16 (9.22) | 18.46 (11.47) | 19.10 (12.08) | 18.23 (10.73) | $<0.001$ |
|  |  |  |  |  |  |  |
| Number of Assessments | 3.47 (1.27) | 3.46 (1.19) | 3.46 (1.11) | 3.43 (1.13) | 3.63 (1.29) | $0.551$ |
| Initial UEMS${}^{1}$ | 29 (24) | 30 (29.5) | 33 (33) | 50 (33) | 28 (40) | $<0.001$ |
| Initial LEMS${}^{2}$ | 32 (23) | 26 (31) | 13 (33) | 0 (0) | 3 (18.5) | $<0.001$ |
| Chronic INCS^3^ | 0.77 (0.19) | 0.63 (0.14) | 0.35 (0.12) | 0.05 (0.08) | -0.29 (0.10) | $<0.001$ |
|  |  |  |  |  |  |  |
| **initial AIS** |  |  |  |  |  | $<0.001$ |
| A | 13 (4.18%) | 52 (6.78%) | 271 (25.46%) | 1533 (70.13%) | 22 (62.86%) |  |
| B | 14 (4.50%) | 93 (12.13%) | 153 (13.81%) | 272 (12.44%) | 5 (14.29%) |  |
| C | 84 (27.01%) | 232 (30.25%) | 313 (28.25%) | 200 (9.15%) | 4 (11.43%) |  |
| D | 200 (64.31%) | 390 (50.85%) | 371 (33.48%) | 181 (8.28%) | 4 (11.43%) |  |
|  |  |  |  |  |  |  |
| **initial NLI** |  |  |  |  |  | $<0.001$ |
| cervical | 250 (80.39%) | 589 (76.79%) | 735 (66.34%) | 959 (43.87%) | 22 (62.86%) |  |
| thoracic | 10 (3.22%) | 46 (6.00%) | 120 (10.83%) | 729 (33.35%) | 6 (17.14%) |  |
| thoracolumbar | 51 (16.40%) | 132 (17.21%) | 253 (22.83%) | 498 (22.78%) | 7 (20.00%) |  |

Table 7 Distribution of patient characteristics, AIS grades and NLI across 5 latent classes.

^1^Upper Extremity Score
^2^Lower Extremity Score
^3^INCS in chronic phase (as determined in table 2, merged overall)
Nominal values and AIS grades in number of patients (percentage), metric values in mean (standard deviation), ordinal values (UEMS and LEMS) in median (interquartilerange)
P-values are calculated using Kruskal Wallis-Test for ordinal/metric values and Chi-square-Test for nominal values
Classes ranked by the mean slope/recovery within each group

Post-hoc testing revealed significant age differences between Classes 1–4, 2–4, and 3–4 ($p<0.001$). Time to first assessment differed significantly between class 1 and all others ($p<0.001$), as well as between classes 2–3 and 2–4 ($p<0.001$). The last assessed INCS in the chronic phase differed between Classes 1–2 ($p<0.05$), 3–5 ($p<0.01$), and all other pairs ($p<0.001$). AIS grades differed across all comparisons ($p<0.001$), except class 1–2 ($p<0.01$) and class 3–5 ($p=0.445$). NLI was significantly different across all classes ($p<0.001$) except 1–2 ($p=0.153$).

For motor scores, UEMS differed between Classes 1–3 ($p<0.01$), 1–5 ($p<0.01$), 2–5 ($p<0.05$), 3–4 ($p<0.01$), and 4–5 ($p<0.01$). LEMS showed highly significant differences for all pairwise comparisons ($p<0.001$), except between Classes 4 and 5 ($p=0.351$).

Supplementary figure 14 Latent class trajectories of neurological recovery based on the INCS for 5 classes: The top panel displays individual patient trajectories color-coded by latent class, overlaid with the class-specific mean recovery trajectories derived from random forest regression (black lines). The lower panels show the respective first derivatives (slopes) and data-driven thresholds (vertical red lines) for the acute, transition, and chronic phases within each class. Class 1 shows the steepest and earliest transition, while class 4 remains largely flat throughout, indicating no recovery. Classes 2 and 3 follow intermediate courses and class 5 which shows a decline. Abbreviations: INCS, Integrated Neurological Change Score; DAI, days after injury

In contrast, a three-class solution collapses the distinctions between recovery patterns, yielding only a group of average recoverers, a group of non-recoverers, and a group of good recoverers (see supplementary figure 15). Patient characteristics, AIS grades and NLI are shown in table 8.

|  | **Class 1** | **Class 2** | **Class 3** | p-value |
| --- | --- | --- | --- | --- |
| Patient Count | 707 | 1329 | 2371 |  |
| Age (years) | 48.50 (18.32) | 50.18 (18.36) | 43.92 (18.73) | $<0.001$ |
| Sex (% male) | 542 (76.66%) | 1040 (78.25%) | 1895 (79.92%) | $0.139$ |
|  |  |  |  |  |
| Initial Assessment | 11.20 (8.25) | 17.30 (11.00) | 19.20 (12.10) | $<0.001$ |
|  |  |  |  |  |
| Number of  Assessments | 3.50 (1.22) | 3.42 (1.12) | 3.44 (1.13) | $0.551$ |
| Initial UEMS${}^{1}$ | 29 (26.5) | 32 (33) | 50 (33.5) | $<0.001$ |
| Initial LEMS${}^{2}$ | 29 (28) | 18 (35) | 0 (0) | $<0.001$ |
| Chronic INCS^3^ | 0.72 (0.16) | 0.41 (0.15) | 0.06 (0.10) | $<0.001$ |
|  |  |  |  |  |
| **initial AIS** |  |  |  | $<0.001$ |
| A | 30 (4.24%) | 253 (19.04%) | 1608 (67.82%) |  |
| B | 411 (58.13%) | 512 (38.53%) | 223 (9.41%) |  |
| C | 212 (29.99%) | 377 (28.37%) | 244 (10.29%) |  |
| D | 54 (7.64%) | 187 (14.07%) | 296 (12.48%) |  |
|  |  |  |  |  |
| **initial NLI** |  |  |  | $<0.001$ |
| cervical | 560 (79.21%) | 928 (69.83%) | 1067 (45.00%) |  |
| thoracic | 33 (4.67%) | 125 (9.41%) | 753 (31.76%) |  |
| thoracolumbar | 114 (16.12%) | 276 (20.77%) | 551 (23.24%) |  |

Table 8 Distribution of patient characteristics, AIS grades and NLI across 3 latent classes.

^1^Upper Extremity Score
^2^Lower Extremity Score
^3^INCS in chronic phase (as determined in table 2, merged overall)
Nominal values and AIS grades in number of patients (percentage), metric values in mean (standard deviation), ordinal values (UEMS and LEMS) in median (interquartilerange)
P-values are calculated using Kruskal Wallis-Test for ordinal/metric values and Chi-square-Test for nominal values
Classes ranked by the mean slope/recovery within each group

Post-hoc testing revealed significant differences in age between Classes 1 and 2 ($p<0.05$), and highly significant differences between Classes 1 and 3 and Classes 2 and 3 ($p<0.001$ each). Time from incident to initial assessment, INCS values in the chronic phase, AIS grade, and NLI all differed highly significantly across all pairwise comparisons ($p<0.001$ for all). In UEMS significant differences were found between Classes 1 and 2 ($p<0.001$) and between Classes 2 and 3 ($p<0.01$), while all pairwise comparisons in LEMS were highly significant ($p<0.001$).

Supplementary figure 15 Latent class trajectories of neurological recovery based on the INCS for 3 classes: The top panel displays individual patient trajectories color-coded by latent class, overlaid with the class-specific mean recovery trajectories derived from random forest regression (black lines). The lower panels show the respective first derivatives (slopes) and data-driven thresholds (vertical red lines) for the acute, transition, and chronic phases within each class. Class 1 shows the steepest and earliest transition, while class 3 remains largely flat throughout, indicating no recovery. Classes 2 follows intermediate courses. Abbreviations: INCS, Integrated Neurological Change Score; DAI, days after injury

However, although the five-class LCA is particularly interesting given the small subgroup of decliners and their characteristics, it is not suitable for further consideration. Since the objective of this work is to calculate a regression curve and identify the relevant thresholds (acute, pre-chronic, and chronic), a subgroup of only 35 individuals is too small to provide reliable estimates. As shown in supplementary figure 13, no chronic thresholds could be defined for class 5. In contrast the three-class LCA provides a more balanced sub-grouping. However, the four-class model offers a finer differentiation between the two average recovery groups (Classes 2 and 3; see 3.2) in terms of their characteristics, as well as UEMS and LEMS scores. For this reason, the four-class model was chosen as the primary solution for the main results which is also coherent with prior work by Badhiwala et al. [37].

## Additional Assessments

Additional assessments are defined as those that do not fall within the predefined EMSCI time frames (very acute = days 1–15; acute I = days 16–40; acute II = days 70–98; acute III = days 150–186; chronic = days 300–546). As shown in supplementary figure 11, assessments outside these intervals are less densely distributed. This raises the question of whether the observed pre-chronic phase is merely the result of patients assessed during these additional time frames (particularly between acute I and acute II as well as acute II and acute III), whether such patients demonstrate better recovery compared to those assessed strictly within the EMSCI time frames (and therefore had an earlier assessment), and whether they differ in certain characteristics that could explain the fluctuations observed in the pre-chronic phase. Patient characteristics can be seen in table 9.

|  | **No Additional** | **Additionals** | p-value |
| --- | --- | --- | --- |
| Patient Count | 3689 | 718 |  |
| Age (years) | 46.78 (18.85) | 45.30 (18.35) | $0.071$ |
| Sex (% male) | 2895 (78.48%) | 582 (81.06%) | $0.133$ |
| Days till Initial Assessment | 17.91 (11.65) | 14.30 (10.50) | $<0.001$ |
| Number of Assessments | 3.28 (1.01) | 4.30 (1.40) | $<0.001$ |
| Initial UEMS${}^{1}$ | 38 (33) | 37 (34) | $0.467$ |
| Initial LEMS${}^{2}$ | 0 (27) | 0 (26.75) | $0.363$ |
| Chronic INCS^3^ | 0.26 (0.28) | 0.3 (0.3) | $0.118$ |
|  |  |  |  |
| **AIS** |  |  | $0.513$ |
| A | 1577 (42.75%) | 314 (43.73%) |  |
| B | 446 (12.09%) | 91 (12.67%) |  |
| C | 701 (19%) | 132 (18.38%) |  |
| D | 965 (26.16%) | 181 (25.21%) |  |
|  |  |  |  |
| **NLI** |  |  | $0.975$ |
| cervical | 2136 (57.90%) | 419 (58.36%) |  |
| thoracic | 764 (20.71%) | 147 (20.47%) |  |
| thoracolumbar | 789 (21.39%) | 152 (21.17%) |  |

Table 9 Distribution of patient characteristics, AIS grades and NLI across patients with and without additional assessments.

^1^Upper Extremity Score
^2^Lower Extremity Score
^3^INCS in chronic phase (as determined in table 2, merged overall)
Nominal values and AIS grades in number of patients (percentage), metric values in mean (standard deviation), ordinal values (UEMS and LEMS) in median (interquartilerange)
P-values are calculated using Kruskal Wallis-Test for ordinal/metric values and Chi-square-Test for nominal values
Classes ranked by the mean slope/recovery within each group

Patient characteristics as well as LEMS, UEMS, AIS Grades and NLI did not differ significantly between groups, except for the timing of the first assessment and the total number of assessments. Patients with additional assessments did, however, show significantly higher INCS in their chronic phase. Thus, patients with additional assessments are not necessarily better recoverers.

# Additional Plots

|   (a) Neurological recovery trajectory based on INCS using LOESS regression. |  |   (b) Neurological recovery trajectory based on INCS using neural network. |
| --- | --- | --- |

Supplementary figure 16 Neurological recovery trajectory based on INCS: scatterplot of INCS values plotted against days after injury, overlaid with the (a) LOESS regression curve and (b) the predicted trajectory from the neural network model (blue lines). The lower panel displays the first derivative (slope) of the smoothed curve, reflecting the rate of neurological change over time. Vertical lines (red) indicate the data-driven thresholds between recovery stages. Abbreviations: INCS, Integrated Neurological Change Score; DAI, days after injury; LOESS, locally estimated scatterplot smoothing

Supplementary figure 17 Neurological recovery trajectory based on INCS using LOESS regression: Scatterplot of all available INCS values plotted against days after injury, overlaid with the LOESS regression curves separately for the AIS groups. Dots represent individual neurological assessments, with lines connecting them to illustrate individual patient trajectories. The upper panel shows the estimated recovery trajectories, while the lower panel displays the first derivative (slope) of the smoothed curves. Vertical lines (red) indicate the data-driven thresholds between recovery stages. Abbreviations: INCS, Integrated Neurological Change Score; LOESS, locally estimated scatterplot smoothing; DAI, days after injury; AIS, American Spinal Injury Association Impairment Scale

Supplementary figure 18 Neurological recovery trajectory based on INCS using LOESS regression: Scatterplot of all available INCS values plotted against days after injury, overlaid with the LOESS regression curves separately for the NLI groups. Dots represent individual neurological assessments, with lines connecting them to illustrate individual patient trajectories. The upper panel shows the estimated recovery trajectories, while the lower panel displays the first derivative (slope) of the smoothed curves. Vertical lines (red) indicate the data-driven thresholds between recovery stages. Abbreviations: INCS, Integrated Neurological Change Score; LOESS, locally estimated scatterplot smoothing; DAI, days after injury; NLI, neurological level of injury

Supplementary figure 19 Neurological recovery trajectory based on INCS using neural network: Scatterplot of all available INCS values plotted against days after injury, overlaid with the predicted trajectory from the neural network model separately for the AIS groups. Dots represent individual neurological assessments, with lines connecting them to illustrate individual patient trajectories. The upper panel shows the estimated recovery trajectories, while the lower panel displays the first derivative (slope) of the smoothed curves. Vertical lines (red) indicate the data-driven thresholds between recovery stages. Abbreviations: INCS, Integrated Neurological Change Score; LOESS, locally estimated scatterplot smoothing; DAI, days after injury; AIS, American Spinal Injury Association Impairment Scale

Supplementary figure 20 Neurological recovery trajectory based on INCS using neural network: Scatterplot of all available INCS values plotted against days after injury, overlaid with he predicted trajectory from the neural network model separately for the NLI groups. Dots represent individual neurological assessments, with lines connecting them to illustrate individual patient trajectories. The upper panel shows the estimated recovery trajectories, while the lower panel displays the first derivative (slope) of the smoothed curves. Vertical lines (red) indicate the data-driven thresholds between recovery stages. Abbreviations: INCS, Integrated Neurological Change Score; LOESS, locally estimated scatterplot smoothing; DAI, days after injury; NLI, Neurological Level of Injury

|    (a) Functional recovery trajectory based on change in the SCIM using LOESS regression. |  |    (b) Functional recovery trajectory based on change in the SCIM using neural network. |
| --- | --- | --- |

Supplementary figure 21 Functional recovery trajectory based on SCIM: Scatterplot of SCIM assessments plotted against days after injury, overlaid with the (a) LOESS regression curve and (b) neural network (blue lines). The lower panel displays the first derivative (slope) of the smoothed curve, reflecting the rate of functional change over time. Vertical lines (red) indicate the data-driven thresholds between recovery stages. Abbreviations: SCIM, Spinal Cord Independence Measure; LOESS, locally estimated scatterplot smoothing; DAI, days after injury

|    (a) Change in hand motor strength using LOESS regression. |  |    (b) Change in hand motor strength using neural network. |
| --- | --- | --- |

Supplementary figure 22 Neurological recovery trajectory based on changes in hand motor strength: Scatterplot of HMS assessments plotted against days after injury, overlaid with the (a) LOESS regression curve and (b) neural network (blue lines). The lower panel displays the first derivative (slope) of the smoothed curve, reflecting the rate of change over time. Vertical lines (red) indicate the data-driven thresholds between recovery stages. Abbreviations: HMS, hand motor strength; LOESS, locally estimated scatterplot smoothing; DAI, days after injury
